# Relapse-related outcomes in the Danish population-based AQP4- antibody seropositive neuromyelitis optica spectrum disorder cohort

**DOI:** 10.1007/s10072-025-08754-y

**Published:** 2026-01-13

**Authors:** Sepehr Mamoei, Sören Möller, Melinda Magyari, Finn Sellebjerg, Jette L. Frederiksen, Kristina B. Svendsen, Rasha Saleem, Helle B. Søndergaard, Anna C. Nilsson, Viktoria Papp, Zsolt Illes

**Affiliations:** 1 Department of Neurology, Odense University Hospital, Odense, Denmark; 2 Department of Neurology, University Hospital of Southern Jutland, Aabenraa, Denmark; 3 Open Patient data Explorative Network, Odense University Hospital, Odense, Denmark; 4 Department of Clinical Research, University of Southern Denmark, Odense, Denmark; 5 Danish Multiple Sclerosis Center and Danish Multiple Sclerosis Registry, Copenhagen University Hospital-Rigshospitalet, University of Copenhagen, Glostrup, Denmark; 6 Department of Clinical Medicine, University of Copenhagen, Copenhagen, Denmark; 7 Department of Neurology, Aarhus University Hospital, Aarhus, Denmark; 8 Department of Clinical Immunology, Odense University Hospital, Odense, Denmark; 9 Department of Molecular Medicine, Odense University Hospital, Odense, Denmark

**Keywords:** Demyelinating diseases, Neuromyelitis optica, Outcome assessment, Time-to-treatment, Disability evaluation

## Abstract

**Background:**

The disease course of AQP4-antibody seropositive neuromyelitis optica spectrum disorder (AQP4-Ab + NMOSD) is unpredictable and variable. We aimed to examine relapse-related outcomes.

**Methods:**

In a nationwide historically prospective AQP4-Ab + NMOSD cohort, relapse outcomes, relapse and maintenance treatments were analyzed by mixed-effects linear and logistic regression models.

**Results:**

Among sixty-six patients (median follow-up: 99.5 months) there were 350 relapses and 272 relapse treatments. Delayed immunosuppression after the first relapse and brainstem (BS) relapses were associated with higher Expanded Disability Status Scale (EDSS) scores at final follow-up. Optic neuritis (ON) was associated with higher relapse frequency. Higher age and male sex were associated with more severe transverse myelitis (TM) (*p* < 0.032), and male sex was also associated with worse ON recovery (*p* = 0.036). ON was more likely after TM (OR: 3.2, 95% CI: 1.7-6.0; *p* < 0.001) and vice versa (OR: 2.9, 95% CI: 1.6–5.2; *p* < 0.001). BS relapse was also more likely after TM (OR: 2.8, 95% CI: 1.2–6.4; *p* = 0.016) and vice versa (OR: 2.4; 95% CI: 1.1–5.3; *p* = 0.031). Rituximab treatment correlated with lower EDSS score at final follow-up (*p* = 0.044).

**Conclusion:**

Earlier initiation of immunosuppressive maintenance treatment limited the accumulation of disability in NMOSD. Recognizing relapse patterns could aid in tailoring monitoring and treatment choices.

## Introduction

Aquaporin-4 antibody seropositive neuromyelitis optica spectrum disorder (AQP4-Ab + NMOSD) is a relapsing autoimmune disease of the central nervous system (CNS) predominantly affecting the optic nerve and spinal cord [1, 2]. Disability in NMOSD evolves due to incomplete recovery between relapses. Five years after disease onset, 22% of AQP4-Ab + patients were reported requiring walking aids, and 41% were blind in at least one eye [3]. This patient group should be considered at risk of relapse indefinitely and in need of permanent immunosuppressive treatment [4]. 

Treatment of NMOSD involves acute relapse treatment aimed at limiting neurological deficits and early initiation of long-term preventive immunosuppressive treatment to minimize relapse frequency and limit accumulation of neurological disabilities [5–8]. Despite trial evidence indicating high efficacy of monoclonal antibody treatments and recent efforts to establish treatment algorithms for patients diagnosed with NMOSD by the International Panel for NMO Diagnosis (IPND) criteria [2, 9, 10], consensus and support of real-world evidences are still required [2, 11]. 

Outcome measures utilized in NMOSD studies usually consist of disability and relapse rate, primarily developed for multiple sclerosis (MS) [8]. The Expanded Disability Status Scale (EDSS) was also adopted from MS to measure disability in NMOSD, despite inadequately estimating prevalent symptoms such as visual function, pain, fatigue, psychiatric and cognitive ability, and upper limb function [4]. 

Studies examining relapse-related outcomes in NMOSD demonstrate that, if untreated, outcomes related to motor and visual functions are poor [12, 13], and the presence of motor symptoms or tetraparesis at first relapse with TM in the first year is a potential predictor of worse long-term EDSS scores [14]. Furthermore, females have a higher risk of relapse than males, people of African heritage have the highest incidence and prevalence, younger patients have a higher likelihood of optic neuritis (ON), higher age at disease onset is associated with a higher frequency of TM and increased likelihood of ambulatory disability, and increasing age is associated with a lower risk of relapse [1, 8, 15, 16]. 

The disease course of NMOSD is variable and heterogeneous, and its dependence on patient/disease characteristics and the combination of relapse treatment and immunosuppressive maintenance treatment is not well understood [8, 17]. Consequently, this population-based national study aimed to examine outcomes of 350 relapses, relapse treatment, immunosuppressive maintenance treatment, and disability measures in patients with AQP4-Ab + NMOSD across all five regions of Denmark.

## Materials and methods

###  Subjects and recruitment

This longitudinal study presents data from a nationwide population-based historically prospective cohort, with data acquired from all AQP4-Ab + adult patients (age ≥ 18) identified from the 9932 patients tested for AQP4-Ab (January 2007 – January 2021) and from the Danish Multiple Sclerosis Registry (DMSR) [18]. The neurological departments at tertiary centers in the four university hospitals at Aalborg, Aarhus, Odense, and Rigshospitalet are treating all patients with NMOSD, referred from the regional neurological departments. Patients in this study were diagnosed with AQP4-Ab + NMOSD according to 2015 IPND criteria [9, 19] and by testing serum for AQP4-Ab with either the commercial fixed cell-based assay (CBA) in Denmark (Euroimmun AG, Lubeck, Germany) or with live CBA at John Radcliffe Hospital, Oxford, UK. Oligoclonal bands were detected by isoelectric focusing followed by immunofixation (Sebia).

###  Variables

Variables were sex, age at onset, number of relapses, type of onset and second relapse, treatment of relapses, severity of the first relapse, time to immunosuppressive treatment, clinicians completed repeated neurostatus EDSS evaluations during the follow-up, and time between first and second relapse. Furthermore, patients were stratified into monophasic and relapsing disease course in order to explore whether the relapse burden correlate with long term accumulation of disability. The severity of the first relapse was categorized for each relapse type. Optic neuritis (ON) severity was categorized as *mild* (visual acuity of 6/12 at nadir), *moderate* (visual acuity between 6/18 and 6/36 at nadir), and *severe* (visual acuity of 6/60 or worse in the affected eye at nadir). Transverse myelitis (TM) severity was categorized as *mild* (walking without aids), *moderate* (walking with aids), and *severe* (unable to walk at nadir). Brainstem relapses (BS) were categorized as *mild* (no aids), *moderate* (in need of aids), and *severe* (respiratory insufficiency). Recovery after the first relapse was categorized as *full*, *partial*, and *none*. Severity and recovery were rated on a scale categorized as 1 (severe and no recovery), 2 (moderate severity/recovery), and 3 (mild severity and full recovery) 6 months after the relapse.

###  Statistical methods

Descriptive statistics was used to assess baseline demographics of the AQP4-Ab + NMOSD population. Normality was assessed using Q-Q plots, histograms, and the Shapiro-Wilks test. Depending on the data distribution, paired t-tests or Wilcoxon sign rank tests were used when comparing monophasic and relapsing disease courses, and Spearman’s rank correlation analysis was utilized as a nonparametric correlation test.

A mixed-effect regression model was utilized as sequential measurements of relapses and relapse types were performed on each patient at various time points. Data analysis was based on univariate, partly adjusted, and fully adjusted models. Odds ratios (OR) were derived from mixed-effects logistic regression models by dichotomizing the occurrence of each type of relapse from the entire follow-up period. Data analysis was based on univariate analysis, partly adjusted analysis examining for confounding, and fully adjusted analysis examined the effect of multicollinearity between independent variables. Mixed-effect regression models were all adjusted for visit, age, sex, severity, and recovery. Annualized relapse rates (ARR) were calculated as the total number of relapses for each patient divided by the observation time in years, excluding patients who were followed up after treatment for less than 6 months [7]. 

STATA/BE 18.0 was used for statistical analysis, with p-values below 0.05 indicating statistical significance. Coefficients from mixed effect regression models are reported with a 95% confidence interval (95% CI), and data in tables are presented as means ± standard deviations (SD).

###  Standard protocol approvals

The Danish Data Protection Agency in the Region of Southern Denmark (file no.: 20/27251) and the Regional Ethical Committee (file no.: S-20200178) approved the study. The Danish Health and Medicines Authority waived the requirement for patient informed consent (file no.: 21/8187).

## Results

Out of the 66 AQP4-Ab + NMOSD patients (90.9% females) with 350 relapses during a median follow-up time of 99.5 months (interquartile range: 39–188 months), 15 (22.7%) had died during follow-up, 17 (25.8%) had a monophasic disease course and 49 (74.2%) had relapses with a median time of 8 months (interquatile range: 3–46 months) between the first and second relapse (Table 1). Compared to patients with a relapsing disease course, patients with monophasic disease had a higher age at the time of diagnosis (55.9 ± 16 vs. 41.2 ± 18.5 years, *p* = 0.005), a shorter observation period (*p* = 0.003) with a median of 40 months (interquatile range 16–100 months) vs. median of 122 (interquatile range 60–239 months), and initiated immunosuppressive maintenance treatment earlier (*p* = 0.009) with a median of 2 months (interquatile range 0.5–3.5 months) vs. median of 13 months (interquatile range 1.75–49.75 months). Patients with relapsing and monophasic disease courses were similar regarding initial EDSS and EDSS score at final follow-up despite the longer observation time in the relapsing group. The groups were also similar in relapse type at disease onset, presence of oligoclonal bands in the cerebrospinal fluid, and number of deceased during follow-up.

**Table 1:** Patient characteristics of patients with AQP4-Ab + NMOSD with monophasic and relapsing disease course

	Total (*n* = 66)	Patients with monophasic disease course (*n* = 17)	Patients with relapsing disease course (*n* = 49)	*p*-value
Female, n (%)	60 (90.9%)	14 (82.4%)	46 (93.9%)	0.154^a^
Age of onset, years (SD) Initial EDSS EDSS at final follow-up Time from first to second relapse, months (SD) Time of follow-up, months (SD) Time to maintenance treatment, months (SD) Number of relapses at final follow up, n (SD) Deceased during follow-up (n %)	45.0 ± 18.9 5.1 ± 2.5 5.1 ± 2.5 - 141.2 ± 147.1 57 ± 131.2 5.1 ± 5.7 15 (22.7%)	55.9 ± 16.0 5.3 ± 3.0 5.6 ± 3.3 - 52.4 ± 42.1 4.0 ± 7.5 1.0 ± 0.0 3 (17.6%)	41.2 ± 18.5 5.0 ± 2.4 5.8 ± 3.1 52.7 ± 100.5 164.7 ± 156.1 72.2 ± 145.3 6.5 ± 6.0 12 (24.5%)	**0.005**^**b**^ 0.901^c^ 0.683^c^ **-** **0.003**^**c**^ **0.009**^**c**^ - 0.487^a^
Ethnicity **-** White, n (%) - Non-white (%)	53 (80,4%) 13 (19.6%)	17 (100.0%) 0 (0.0%)	36 (73.6%) 13 (26.4%)	**0.018**^**a**^
Relapse type at disease onset - Optic neuritis, n (%) - Transverse myelitis, n (%) - Brainstem and area postrema syndrome, n (%) - Multifocal presentation - Other*	20 (30,3%) 32 (48,5%) 5 (7.5%) 6 (9.1%) 3 (4.5%)	4 (23.5%) 9 (52.9%) 1 (5.9%) 2 (11.8%) 1 (5.9%)	16 (32.7%) 23 (46.9%) 4 (8.2%) 4 (8.2%) 2 (4.1%)	0.481^a^ 0.670^a^ 0.773^c^ 0.656^a^ 0.759^a^
Examined for oligoclonal bands, n Positive oligoclonal bands at first relapse, n (%)	*n* = 51 11 (21.6%)	*n* = 13 2 (15.4%)	*n* = 38 9 (23.7%)	0.530^a^

*AQP4* aquaporin-4, *NMOSD* neuromyelitis optica spectrum disorder, *n* number, *SD* standard deviation, * symptomatic cerebral symptom or diencephalon, ^a^: chi-squared-test; ^b^: unpaired t-test; ^c^: Mann-Whitney U test. The bold *p*-values signify statistical significance

###  Time to immunosuppressive maintenance treatment and its association with the EDSS

**Fig. 1:**
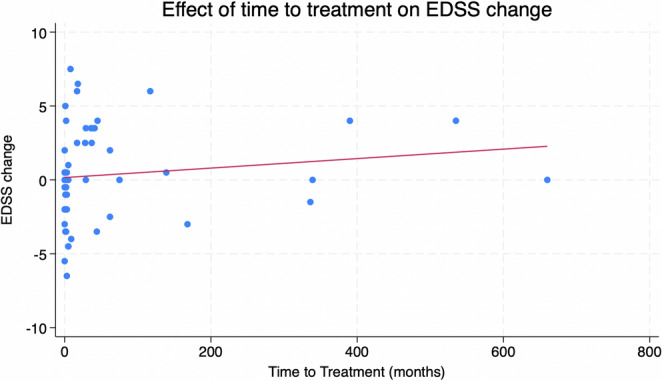
Association between the change in EDSS between first visit and final follow-up and the time to maintenance treatment after the first relapse

Longer time between the first relapse and initiation of immunosuppressive maintenance treatment (mean 57 ± 131.2 months) correlated with higher increase in EDSS scores throughout the entire follow-up period (*p* = 0.025, Spearman’s rho 0.300) and a higher EDSS score at the final follow-up (*p* = 0.013, Spearman’s rho 0.308) (Fig. 1). Longer time between the first relapse and initiation of maintenance treatment was also associated with higher number of relapses (*p* = 0.016, Spearman’s rho 0.475). Time between first and second relapse with a median of 8 months (interquatile range: 3–46 months) was not associated with the EDSS score at the final follow-up (*p* = 0.170, Spearman’s rho 0.223).

###  Annualized relapse rates and relapse types

Excluding patients with less than 6 months of follow-up, annualized relapse rate (ARR) was assessed in 59 patients (32 had ON, 52 had TM, and 20 had brainstem relapses). Three patients were followed up after 6 months, and 4 patients were only followed up once. The median number of observed relapses for each patient was four (range, 1–29). The median ARR for all documented relapses was 0.48 (range, 0.07–2.29, mean 0.67). The median ARR for ON was 0.19 (range, 0.04–2.25, mean 0.36), for TM 0.35 (range, 0.02–2.18, mean 0.48), and for BS relapses 0.16 (range, 0.03–0.77, mean 0.22).

###  Type of initial relapse and subsequent relapses

The majority of initial relapses during both the monophasic and relapsing disease courses (Table 1) were TM (52.9% and 46.9%, respectively), followed by ON (23.5% and 32.7%, respectively). An initial relapse of ON was the only relapse type that correlated with a higher number of subsequent relapses (*p* = 0.034, Spearman’s rho 0.261). The mixed-effects logistic regression models showed that ON was more likely after TM (OR: 3.2, 95% CI: 1.7–6.0.7.0; *p* < 0.001) and vice versa (OR: 2.9; 95% CI: 1.6–5.2; *p* < 0.001). BS relapse was also more likely after TM (OR 2.8, 95% CI: 1.2–6.4; *p* = 0.016) and vice versa (OR: 2.4; 95% CI: 1.1–5.3; *p* = 0.031).

###  Relapses and their effects on disability

Number of relapses during the follow-up period did not correlate with the EDSS score at the final follow-up (*p* = 0.114, Spearman’s rho 0.203). The mixed-effects regression model, which encompassed disability assessments (EDSS) and the total number of relapses in the entire follow-up period for each patient, demonstrated that BS relapses were associated with a higher EDSS score throughout the follow-up period (Table 2) in the univariate and partly adjusted analysis (*p* < 0.001). The number of ON and TM relapses was not associated with EDSS throughout the follow-up period (*p* > 0.05).

**Table 2:** Mixed-effects regression models and associations between relapse type, disability, severity, and recovery after initial relapse

		Univariate		Partially adjusted		Fully adjusted	
Outcome	Variable	Coefficient (95% CI)	*p*-value	Coefficient (95% CI)	*p*-value	Coefficient (95% CI)	*p*-value
Disability							
EDSS	ON TM BS	0.00 (−0.17; 0.17) 0.13 (−0.02; 0.29) 0.57 (0.24; 0.89)	0.991 0.097 **0.001**	−0.05 (−0.23; 0.12) 0.12 (−0.05; 0.28) 0.54 (0.21; 0.87)	0.554 0.167 **0.001**	−0.37 (−0.99; 0.24) 0.56 (−0.25; 1.38) 1.29 (−0.13; 2.71)	0.234 0.175 0.075
Relapse Severity							
ON	Age Sex	0.00 (−0.01; 0.02) −0.28 (−1.30; 0.73)	0.781 0.586	0.00 (−0.1; 0.02) −0.27 (−1.29; 0.75)	0.822 0.604	0.00 (−0.02; 0.02) −0.28 (−1.31; 0.74)	0.859 0.592
TM	Age Sex	−0,01 (−0.02; −0.00) −1.00 (−1.91; −0.09)	**0.021** **0.032**	−0.01 (−0.02; 0.00) −0.79 (1.70; 0.11)	0.056 0.087	−0.01 (−0.02; 0.00) −0.62 (−1.50; 0.26)	0.099 0.168
BS	Age Sex	−0.02 (−0.04; 0.01) 1.00 (0.85; 2.67)	0.194 0.241	−0.02 (−0.04: 0.00) 1.25 (−0.27; 2.79)	0.085 0.106	−0.01 (−0.03; 0.01) 0.75 (−0.50; 2.01)	0.176 0.238
Relapse Recovery							
ON	Age Sex	0.01 (−0.01; 0.02) −1.08 (−2.08; −0.09)	0.451 **0.033**	0.01 (−0.01; 0.02) −1.06 (−2.04; −0.07)	0.524 **0.036**	0.00 (−0.01; 0.02) −1.07 (−2.06; −0.08)	0.564 **0.035**
TM	Age Sex	−0.01 (−0.02; 0.00) −049 (−1.34; 0.36)	0.290 0.258	−0.00 (−0.02; 0.01) −0.4 (−1.27; 0.46)	0.409 0.360	−0.00 (−0.02; 0.01) −0.44 (−1.33; 0.45)	0.395 0.330
BS*	Age Sex	0.00 (−0.01; 0.02) −0.55 (−1.52; 0.43)	0.740 0.273	0.00 (−0.01; 0.02) −0.60 (−1.58; 0.38)	0.564 0.230	0.00 (−0.01; 0.01) −0.35 (−1.25; 0.55)	0.913 0.446

*Abbreviations: EDSS* Expanded Disability Status Scale, *95% CI*, 95% Confidence interval, *ON* optic neuritis, *TM* transverse myelitis, *BS* brainstem. *also includes area postrema syndrome

###  Age association with relapse severity and recovery after the first relapse

The mixed-effects regression model that included assessments of recovery after the first relapse, age, and sex demonstrated that male sex (*p* = 0.032) and higher age (*p* = 0.021) were associated with more severe relapses of TM in the univariate analysis (Table 2). Male sex was also associated with worse recovery after ON in the univariate (*p* = 0.033), partly adjusted (*p* = 0.036), and fully adjusted (*p* = 0.035) analyses.

###  Relapse treatment and immunosuppressive maintenance treatment

The total number of relapses (*n* = 350) comprised 97 ON relapses, 215 TM relapses, 38 BS relapses (including area postrema syndrome, *n* = 3), and excluding symptomatic cerebral syndrome (*n* = 2). A total of 272 relapses were treated (relapse treatment unknown for 11 relapses), while 78 relapses were not treated; 46 of these occurred before 2007, when patients were not diagnosed with AQP4-Abs. Relapses were treated with oral methylprednisolone (OMP, *n* = 46), intravenous methylprednisolone (IVMP) (*n* = 150), plasma exchange (PLEX) (*n* = 3), and combination therapies consisting of IVMP/PLEX (*n* = 44), IVMP/PLEX/intravenous immunoglobulins (IVIG) (*n* = 6), IVMP/IVIG (*n* = 2), OMP/IVMP/PLEX (*n* = 9), and IVIG/PLEX (*n* = 1). Relapse treatment with PLEX and the combination therapies, except for IVMP/PLEX, were not included in the regression analysis because of the low number.

Linear regression models demonstrated that not receiving relapse treatment correlated with a higher EDSS score at the final follow-up (coefficient 0.65, 95% CI [0.08; 1.21], *p* = 0.025). In contrast, the same model showed that relapse treatment with IVMP (*p* = 0.109) and IVMP/PLEX (*p* = 0.867) was not associated with a higher EDSS score at the final follow-up.

Of the total 350 relapses recorded in the cohort, 198 occurred while patients were untreated, 150 occured during maintenance treatment, and 2 occured during periods with unknown treatment status. Among the patients receiving maintenance treatment; 44 patients treated with azathioprine (median duration 10.8 months, interquartile range: 3.3–73.0 months) had 49 relapses; 24 patients treated with rituximab (median duration 29.7 months, interquartile range 14.8–57.2 months) had 11 relapses; 32 patients treated with prednisolone (median duration 11.1 months, interquartile range 5.8–31 monts) had 16 relapses; 11 patients treated with methotrexate (median duration 27.5 months, interquartile range 7.8–48.5 months) had 7 relapses; 8 patients treated with interferon-beta/glatiramer acetate (median duration 14.1 months, interquartile range 8.1–37.3 months) had 25 relapses; 4 patients treated with ofatumumab (median duration 36.7 months, interquartile range 26.0–47.2 months) had 5 relapses. 4 patients with IVIG treatment (median duration 8.7 months, interquartile range 6.5–8.9 months) did not have relapses. Because of the low number of patients (≤ 3), we did not evaluate relapses during treatment with tocilizumab; mycophenolate mofetil, mitoxantrone; cyclophosphamide; or cyclosporine.

Three patients in the entire cohort had > 20 relapses of which a majority (>16 relapses) occurred before the availability of AQP4-IgG testing in 2007, while being treated with mainly interferon-beta.

Of the 17 patients in the monophasic group, 14 were diagnosed after 2012, who were treated with methotrexate (*n* = 2), azathioprine (*n* = 4), ofatumumab (*n* = 1), rituximab (*n* = 6), and one with unknown treatment. Three patients in the monophasic group, had disease onset before the availability of AQP4-IgG testing in 2007, of which one received azathioprine and the remaining did not receive any immunosuppressive treatment.

Treatment with rituximab correlated with a lower EDSS score at the final follow-up in both linear regression models (coefficient − 2.35, 95% CI [−4.18; −0.52], *p* = 0.013) and Spearman’s rank correlation analysis (*p* = 0.044, Spearman’s rho − 0.257). Linear regression analysis comparing azathioprine and rituximab demonstrate that rituximab (coefficient − 1.65, 95% CI [−3.27; −0.04], *p* = 0.045) is correlated with a lower EDSS score at the final follow unlike azathioprine (coefficient 0.58, 95% CI [−1.09; 2.25], *p* = 0.488).

## Discussion

Using a nationwide population-based historically prospective cohort, we analyzed 350 relapses and 243 relapse treatments in 66 adult patients with AQP4-Ab + NMOSD. The relatively small sample size may have limited the statistical power of the study; therefore, the results should be interpreted as exploratory.

###  Initial relapse and initiation of immunosuppressive maintenance treatment

Our data showed that initial relapses of TM were the most frequent, followed by ON, multifocal relapses, and BS relapses in patients with both a monophasic and relapsing disease course, similar to the findings of other retrospective cohort studies [12, 14]. The shorter follow-up period in monophasic patients can be attributed to the older age at initial relapse and severe onset relapse leading to death. The correlation between a longer time to immunosuppressive maintenance treatment after the initial relapse and higher EDSS score highlight the importance of early initiation of immunosuppressive treatment to minimize the frequency of relapses [5, 7, 10, 17]. A substantial proportion of our patients did not receive such therapy, because the data were derived from a historical cohort treated before the adoption of more standardized treatment strategies. The importance of early initiation of immunosuppressive maintenance treatment is also underlined by the increased mortality and shorter life expectancy in the same cohort with the cause of death directly linked to the disease in 93% of cases [20]. 

### Relapse patterns*,* disability, and relapse recovery

AQP4-Ab + NMOSD is characterized by an unpredictable pattern of relapses [21–24]. We found that patients with TM were more likely to experience ON or BS relapses and, to a lesser degree, the other way around. BS relapses were associated with higher EDSS scores throughout the follow-up period. In a previous study, brainstem atrophy, especially reduced volume of the medulla oblongata correlated with a higher EDSS score in patients with NMOSD [25]. A retrospective study of 170 patients with NMOSD demonstrated that patients with lesions in the medulla oblongata had higher ARR and EDSS scores, attributed to the higher co-occurrence of supra- and infratentorial lesions alongside the brainstem lesions, while the symptoms of brainstem lesions such as paresis, dysphagia, nystagmus, and dysarthria potentially affecting the EDSS score more severely [26]. The lack of association between the number of TM and disability could possibly be attributed to some patients from the cohort dying shortly after disease onset and not having more than one visit in the outpatient clinic. In a mortality study of the same cohort, an increased mortality was shown with all who died having at least one longitudinally extensive TM [20]. Severe early TM may also cause such early disability that additional TMs could contribute little along the course, and this may be another factor to explain lack of association between disability and the number of TM relapses. Furthermore, the lack of association with ON and disability could further highlight that visual impairment contributes little to the EDSS, possibly overshadowed by high EDSS due to high ambulation scores, as found in another study following neurological impairment 10 years after ON [27]. 

A total of 3 patients presented with > 20 relapses, of which a majority occurred before the availability of AQP4-IgG testing and were treated with interferon-beta because of misdiagnosis of multiple sclerosis. Interferon-beta therapies are known to increase relapse activity in NMOSD [28]. On the other hand, a majority of the monophasic group were diagnosed after 2012, when early initiation of maintenance treatment became standard practice and relapse activity was relatively well controlled with conventional immunosuppressive treatments and rituximab.

ARR was lower compared to other studies [3, 5, 7, 14, 29], which can be explained by the higher proportion of patients with a monophasic disease course in our cohort (25.8%) when compared to other cohorts (12.2% and 6%) [12, 14]. Furthermore, in patients diagnosed more recently, the treatment delay is also minimized, which is also evident in the patients with a monophasic disease course, who initiated immunosuppressive maintenance treatment earlier than patients with a polyphasic disease course.

EDSS scores remained comparable between the patients with monophasic and relapsing disease courses, despite monophasic patients starting immunosuppressive maintenance earlier. However, regression analysis of the entire cohort demonstrated an association between longer time to initiation of immunosuppressive maintenance treatment and higher EDSS scores. Patients from the monophasic disease course were older, had more severe TM relapses at disease onset. This may explain the similar EDSS score, compared to the patients with a relapsing disease course, despite beginning immunosuppressive treatment earlier. The association between higher age and more severe TM relapses could signify an age-dependent diminished restorative capacity of the CNS and reduced response to treatment [30, 31]. An age-dependent reduced chance of complete recovery after a relapse (24% reduction for each decade) and TM in late-onset NMOSD, has also been demonstrated in patients with AQP4-Ab + NMOSD [12, 31]. 

We found that male sex was associated with more severe TM relapse and worse recovery after first ON relapse. Interpreting this finding was challenged by the significantly lower prevalence of male patients (9.1%) in the cohort compared with female patients (90.9%). Studies of sex differences in TM relapse patterns present conflicting results [32–34]. A large retrospective cohort study found different relapse patterns in females and, unlike males, a peak in relapses at 30–40 years [31]. 

###  Immunosuppressive maintenance treatment and disability

Immunosuppressive maintenance treatment is essential in reducing inflammation and preventing the accumulation of neurological deficits. Azathioprine, rituximab, and mycophenolate mofetil are the most frequently used off label immunosuppressive maintenance treatments in NMOSD [1]. Our results demonstrated that maintenance treatment with rituximab was correlated with a lower EDSS score at the final follow-up. Several studies have also demonstrated the beneficial effects of rituximab in reducing the frequency of relapses and lower EDSS scores [7, 35–38], and a small randomized clinical trial suggested that rituximab was effective in preventing relapses in patients with AQP-Ab + NMOSD [39]. However, unaccounted confounders, and other overlapping therapies may limit the strength of this conclusion. It should also be noted that rituximab was approved as immunosuppressive maintenance treatment for AQP4-Ab + NMOSD in Japan in 2022. Randomized clinical trials with additional novel monoclonal antibodies such as eculizumab, inebilizumab, satralizumab, and ravulizumab showed high efficacy [1, 10, 39]. Furthermore, the efficacy of mycophenolate was comparable to rituximab in another study, whereas the risk of relapse was more than twofold higher when azathioprine was compared to rituximab [7, 10, 15, 40–42]. 

###  Relapse treatment and recovery

The choice of relapse treatment was heterogeneous, with most relapses treated with IVMP, followed by OMP, IVMP/PLEX, and other combinations of relapse treatments. EDSS scores in the follow-up period did not increase when relapses were treated. However, untreated relapses were associated with higher EDSS scores in the follow-up period, signifying a higher degree of disability. The high number of untreated relapses in this cohort can be explained by patients being misdiagnosed before testing for AQP4-Ab and meeting the IPND criteria. This finding again highlights the importance of relapse treatment in NMOSD to reduce the accumulation of disability over time [43]. Other studies have also suggested that without relapse treatment, recovery is poor with very few regaining full recovery [12, 14]. Standard relapse treatment at our institutions was based on corticosteroids and the subsequent escalation with PLEX if recovery was insufficient, which also is in line with retrospective studies recommending a combination of corticosteroids and PLEX with minimal delay [44, 45]. 

###  Conclusion

In conclusion, analysis of 350 relapses and 243 relapse treatments in this national cohort study of 66 patients showed relapse patterns in patients with AQP4-Ab + NMOSD, which could potentially aid clinicians in tailoring patient monitoring and treatment choice. Conclusions about certain immunosuppressive maintenance treatments are exploratory due to the limited number of patients. However, our data indicated that earlier initiation of immunosuppressive maintenance treatment, particularly with a highly efficient monoclonal antibody could limit the accumulation of disability over time.

## Data Availability

The raw data supporting the conclusions of this article will be made available by the authors, without undue reservation.
